# Chevalier's Drill Stock

**Published:** 1851-07

**Authors:** 


*Chevalier's Drill Stock.*—Since writing the notice of this instrument, we have received a very beautifully finished one, and find it useful. For many operations, such an instrument is indispensable.

